# Aqueous Two-Phase System Extraction of Polyketide-Based Fungal Pigments Using Ammonium- or Imidazolium-Based Ionic Liquids for Detection Purpose: A Case Study

**DOI:** 10.3390/jof6040375

**Published:** 2020-12-18

**Authors:** Juliana Lebeau, Thomas Petit, Mireille Fouillaud, Laurent Dufossé, Yanis Caro

**Affiliations:** 1 Laboratoire de Chimie et de Biotechnologie des Produits Naturels (CHEMBIOPRO), Université de La Réunion, F-97490 Sainte-Clotilde, France; juliana.lebeau@gmail.com (J.L.); thomas.petit@univ-reunion.fr (T.P.); mireille.fouillaud@univ-reunion.fr (M.F.); laurent.dufosse@univ-reunion.fr (L.D.); 2 Département Hygiène Sécurité Environnement (HSE), Université de La Réunion—IUT La Réunion, F-97410 Saint-Pierre, France

**Keywords:** fungal pigment, natural colorant, extraction ability, marine fungi, *Talaromyces albobiverticillius*, aqueous two-phases system extraction, ionic liquids

## Abstract

Demand for microbial colorants is now becoming a competitive research topic for food, cosmetics and pharmaceutics industries. In most applications, the pigments of interest such as polyketide-based red pigments from fungal submerged cultures are extracted by conventional liquid–liquid extraction methods requiring large volumes of various organic solvents and time. To address this question from a different angle, we proposed, here, to investigate the use of three different aqueous two-phase extraction systems using either ammonium- or imidazolium-based ionic liquids. We applied these to four fermentation broths of *Talaromyces albobiverticillius* (deep red pigment producer), *Emericella purpurea* (red pigment producer), *Paecilomyces marquandii* (yellow pigment producer) and *Trichoderma harzianum* (yellow-brown pigment producer) to investigate their selective extraction abilities towards the detection of polyketide-based pigments. Our findings led us to conclude that (i) these alternative extraction systems using ionic liquids as greener extractant means worked well for this extraction of colored molecules from the fermentation broths of the filamentous fungi investigated here; (ii) tetrabutylammonium bromide, [N4444]Br-, showed the best pigment extraction ability, with a higher putative affinity for azaphilone red pigments; (iii) the back extraction and recovery of the fungal pigments from ionic liquid phases remained the limiting point of the method under our selected conditions for potential industrial applications. Nevertheless, these alternative extraction procedures appeared to be promising ways for the detection of polyketide-based colorants in the submerged cultures of filamentous fungi.

## 1. Introduction

Improving sustainability and reducing the amounts of waste produced and discarded by chemical industries is a global challenge. The production of dyes is one of the industries which is tackling these issues, aiming to minimize these impacts by using viable and beneficial alternatives to both human health and the environment. The demand for natural colorants has been growing over the past two decades and is now becoming a competitive research topic for food, cosmetics, pharmaceutics and textiles industries [1]. The dyeing industry is currently suffering from the rising cost of raw materials due to the increasing demand for eco-friendly pigments to replace synthetic dyes (such as azo dyes). This is particularly true for the red-dye industries, which have no, or very few, natural red pigment alternatives with an adequate robustness meeting both the organoleptic expectations of consumers and the requirements of industrial processes. Unlike synthetic dyes, natural colorants are environmentally friendly as they do not require heavy and time- and energy-consuming benzene-based chemical synthesis, nor are they generating pollution and toxicity to human health wastewaters. Moreover, natural dyes often come as liquid solutions, while synthetic dyes tend to be dry powders, consequently requiring large amounts of water for dying purposes [2].

Recent literature has underlined the need to investigate new bio-pigments derived from bio-sources and notably pigments from microorganisms [3]. Indeed, the positive biological properties of some natural dyes, such as carotenoids and polyketide-based pigments of microbial origin, have made it possible to widen the potential area of specific applications of these natural colorants (such as for functional finishing of textiles, in functional foods, pharmaceuticals, etc.) [3]. Among unconventional sources, ascomycetous fungi of either marine or terrestrial origin are known to produce an extraordinary range of biomolecules such as bioactive polyketide pigments, which often tend to be more stable and water-soluble than plant pigments [4]. Thus, fungal colored polyketides are the most promising material in this respect. The production of natural colorants with a polyketide structure from filamentous fungi has already been reported, mostly using *Monascus, Talaromyces, Emericella, Paecilomyces* and *Trichoderma* species [3,4]. These aforementioned fungal species produce polyketide pigments with various hues, from yellow to reddish-purple, in the mycelium or as diffusing colorants in the fermentation broth (submerged culture). These fungal metabolites are structurally related (i.e., are biosynthesized by polyketide synthases, PKS) and are either azaphilone, naphthoquinone or hydroxyanthraquinone polyketide-based pigments [4].

However, to fully meet the demand of natural red colorants for the industries, there is an urgent need to develop and design green and sustainable detection, extraction and isolation techniques with higher recovery and greater selectivity in a faster and more benign manner than conventional methods. Indeed, the natural pigments available from plant, insect or microbial origins are currently essentially extracted using time-consuming, conventional liquid–liquid extraction methods that also require large volumes of various organic solvents. These conventional techniques usually involve prolonged contact with excess of organic solvents or a mixture thereof under high temperatures and/or pressures [5]. Moreover, these techniques sometimes require toxic solvents such as chlorinated or halogenated solvents, such as dichloromethane or toluene [6], and may impact on the final application (food, cosmetics, textiles, etc.) of the extracted colorants in regards to health and safety concerns. Furthermore, they often result in a relatively low extraction yield, poor selectivity and the need for further energy-intensive purification and wastewater recycling steps. Thus, any large-scale application raises environmental concerns, as well as uncertainties about their economic viability. Both scale-up and cost-effectiveness of the production of natural pigments at the industrial level, with a particular emphasis on red fungal pigments for food, cosmetic, pharmaceutical and fabric applications, still remain limited and require substantial optimization [4]. Ionic liquids, which are considered in this study, are new, unconventional solvents that have been receiving growing attention since the early 2000s because of their potential as green solvents in addition to their properties that are tunable to the target products and recyclability [7,8,9,10,11,12,13].

The aim of this study is the investigation of the potential of ionic liquids (ILs) as a more sustainable extraction technique for detection purpose of polyketide pigments from fermentation broths of filamentous fungi. Thus, we investigated the use of three different ammonium- or imidazolium-based ionic liquids, namely tetrabutylammonium bromide ([N4444]Br^−^), 1-butyl-3-methylimidazolium bromide ([C4Mim]Br^−^) and 1-butyl-3-methyl-imidazolium chloride ([C4Mim]Cl^−^), as new types of solvents in aqueous two-phase system (ATPS) extraction. An IL-ATPS is formed by mixing a soluble IL (chaotropic agent) and a salt with a kosmotropic profile (Na_2_CO_3_ and Na_2_CH_3_CO_2_). These systems allow the separation of two water-soluble compounds into two immiscible aqueous-rich phases. These specific ILs and salts were selected based on their water-miscibility profiles as well as their safety. It is worth mentioning that IL-based systems exhibit varying degrees of eco-toxicities driven both by the aromatic/alkyl chain of the IL itself as well as by the type of anionic salt it has been combined with [7,14,15]. As an example, tetrafluoroborate and hexafluorophosphate anions have been the commonly used salts with ILs due to their ability to enhance phase stability among other physicochemical advantages. However, fluorinated-based salts have the deleterious tendency to be hydrolyzed into toxic hydrofluoric acid [15]. Similarly, even the most hydrophobic ILs can be partially dispersed in water and become a potential polluting agent if not closely monitored. Only a few studies have addressed the environmental impact or potential risks associated with ILs’ design, use and recycling. Thus, a rigorous selection of ILs and salt families and structures must be carried out beforehand based on the type of target biomolecules to be extracted, in order to make the most sustainable choice [14,16,17,18]. Imidazolium-based ILs such as [C4Mim]Br^−^ and [C4Mim]Cl^−^ are the most frequently used IL cores and are, therefore, well documented [19,20], hence we chose to study these in association with harmless salts. They were proven to have higher hydrophilicity than tetra-alkyl ammonium-based ILs ([N4444]Br^−^) because of their aromatic characteristics allowing a stronger interaction with water due to the delocalization of the positive charge over the aromatic ring [21]. However, the combination with anions and kosmotropic salt also has a significant impact on the final ability to form ATPS [22,23,24], as well as on the polarity of the overall system, and thus potentially influences the final partition of fungal pigments. Moreover, the extraction capabilities of the IL-based system will be challenged by the fact that the target fungal pigments are contained in fermentation broths. These are complex biological matrices containing a myriad of other components such as proteins, lipids and other excretable secondary metabolites, which can most likely impact the chemical structure and availability of the target compounds. Therefore, both hydrophilic and hydrophobic IL cations combined with ATPS-forming anions and organic and inorganic salts were investigated here.

Within this scenario, we investigated this alternative IL-ATPS extraction technique using the three aforementioned ammonium- or imidazolium-based ionic liquids to selectively extract the pigmented polyketides from submerged cultures of four filamentous fungi producing a mixture of pigments, namely *Talaromyces albobiverticillius* (producing deep red pigments), *Emericella purpurea* (producing red pigments), *Paecilomyces marquandii* (producing yellow pigments) and *Trichoderma harzianum* (producing yellow to brown pigments). These four strains were selected because they are well known to produce biomolecules exhibiting a rich chemical and structural diversity with different colors, including red ones [25,26]. *Talaromyces* represents a good example of the diversity of fungal pigments. In our previous study, we highlighted the potential of the marine-derived fungus *T. albobiverticillius* 30548 to produce, in a submerged culture, azaphilone polyketide-based red colorants, such as *N*-threonine-monascorubramine, *N*-glutaryl-rubropunctamine and PP-O [26]. In other previous studies, some azaphilone (hexaketide) compounds with biological activities have also been isolated from the fermentation broth of *T. harzianum*, such as the yellow azaphilone pigment T22 [27] and the compounds harziphilone and fleephilone [28], with some other extrolites with different chemical structures (cyclonerodiol, trichosetin, harzianic acid, harzianopyridone and MR566A) [29]. Some species of *Trichoderma* are natural producers of anthraquinone (octaketide) pigments, whose biological activities are also well known. For example, several anthraquinone pigments have been reported earlier from a fermentation broth of *T. harzianum*—crysophanol (red), pachybasin (orange), emodin (orange), ω-hydroxypachybasin, 1,5-dihydroxy-3-hydroxymethyl-9,10-anthraquinone, 1,7-dihydroxy-3-hydroxymethyl-9,10-anthraquinone, 1,8-dihydroxy-3-methylanthraquinone and 1-hydroxy-3-methylanthraquinone [30,31]. Catenarin (red) is an anthraquinone derivative that has been isolated from a variety of fungi, including species of *Emericella* [4]. *Emericella* sp. is also known for its ability to produce other bioactive secondary metabolites with different chemical structures, including polyketide pigments. In particular, various extrolites such as epurpurin A–C, variecolins, versicolorins, albistrins, emerin, emindol PA, norsolorinic acid and shamixanthones have been isolated from *Emericella purpurea* [32]. Next, sorbicillinoids (e.g., sorbicillin, sorbicillinol, oxosorbicillinol and bisvertinol) are an important family of hexaketide compounds produced by terrestrial and marine fungi, including species of *Trichoderma* (e.g., *T. harzianum*) and *Paecilomyces* (e.g., *P. marquandii*) [33].

In regards to the diversity of the expected compounds, the selectivity of the IL-ATPS extraction for specific types of fungal pigments (e.g., red ones) was investigated. Thus, the main goal of our study here is the selective extraction of the fungal polyketide-based pigments with a red hue from the fermentation broths. Additionally, we looked into the reverse extraction of the colored molecules from the IL-ATPS by working on the ionizable profile of the polyketide molecules. Ultimately, this gave us tools to estimate the overall rate of pigment recovery from the fermentation broth and identify points of future improvements. It is worth mentioning that most of the studies conducted so far using IL-based extraction methods on biomaterials (fungal-, plant- or insect-originated) explored only either the extraction power of the IL on pure solution of compounds of interest or the stability and/or potential use of the said compounds in the IL phase [34]. Few and infrequent studies have been conducted on testing the recovery of the molecule of interest from the IL-based extractant as well as its reuse [34]. Nevertheless, it may be noted that the accurate composition of all the secondary metabolites produced by these aforementioned fungal strains has not been investigated here, because that is not the issue.

## 2. Materials and Methods

### 2.1. Selection of the Ionic Liquids and Formation of the Kosmotropic Salt Buffer

The compositions and structures of the three ionic liquids, ([N4444]Br^−^), ([C4Mim]Br^−^ and ([C4Mim]Cl^−^), used in this study are presented in Table 1. The reagents were purchased from Sigma Aldrich (Saint Louis, MO, USA). Alizarin (anthraquinone) purchased from Sigma Aldrich was used as control for IL-ATPS extraction purposes.

### 2.2. Fungal Strains and Culture Conditions

**Figure 1:**
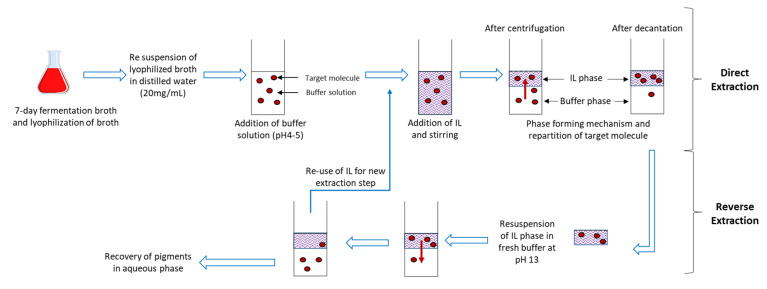
Brief description of the overall ideal IL aqueous two-phase system (IL-ATPS) workflow for direct detection and extraction of fungal pigments into ionic-rich phase, their back-extraction into salt-rich solution and re-injection of IL into the process.

The marine-derived fungal strain *T. albobiverticillius* (collection number 30548, GenBank accession number MK937814) was isolated from the back reef-flat of Réunion Island [35]. The three other strains used in this study were isolated from terrestrial environments, including *T. harzianum* (commercial biological control strain T22), *E. purpurea* (collection reference LCP 3323) and *P. marquandii* (collection reference LCP 2271) and were bought from the fungal culture collection of the Muséum national d’Histoire naturelle (MNHN, Paris, France). For submerged cultures, potato dextrose broth (PDB; Sigma Aldrich, Saint Louis, MO, USA) was used routinely as culture medium. The pH of the culture medium was adjusted to 6.0 ± 0.2 using 0.1 M HCl prior to sterilization. Pre-culture and cultivation were carried out in 250 mL Erlenmeyer flasks containing 100 mL of sterilized culture medium. The flasks were incubated at 26 °C for 7 days and agitated at 150 rpm using a rotary agitator (Infors Multitron, 50 mm excentration, Infors HT, Switzerland) as described by Lebeau and coworkers (2017) [25]. After 7 days of fermentation, the contents of each flask were collected and centrifuged at 14,000× *g* for 10 min; the resulting supernatant was filtered through a Whatman filter paper (GF/C) at a reduced pressure using a Büchner funnel to obtain the culture filtrate. The filtered colored supernatants were then freeze-dried at −84 °C in an ultra-low-temperature freezer (Sanyo, Guangzhou, China) for at least 2 h. The samples were then quickly transferred to a LABCONCO FreeZone 2.5 lyophilizer (LABCONCO, Kansas City, MO, USA) and lyophilized for 24 h. During freezing, the condenser temperature and vacuum pressure were maintained at −47 °C and 200 mbar, respectively (Figure 1).

### 2.3. Binodal Curves Determination

Ternary phase diagrams for each of the IL-ATPS-forming systems were determined using the Cloud Point titration method at room temperature and pressure [36]. Aqueous solutions of salts at 40% *w*/*w* and aqueous solutions of ILs at 30% *w*/*w* were also prepared at room temperature and pressure and were used for the determination of the binodal curves for each IL-ATPS. It was carried out by the drop-wise addition of the aqueous solution of the concentrated salt to the IL aqueous solution (or pure polymer) until the detection of a cloudy and biphasic solution. This was followed by the drop-wise addition of pure water (diluent) until the formation of a clear and limpid solution corresponding to the monophasic regime. The composition of the two-phase system was determined using the ratio of the weight of one component added to the total weight of all added components. The above procedure was repeated until the least amount of turbidity was observed to obtain sufficient data to generate binodal curves [37,38]. At the level of the biphasic region, the upper phase was composed by the IL-rich aqueous phase while the lower phase was the salt-rich phase for all ILs and salts investigated in this study.

### 2.4. Fungal Pigments Partitioning Using Ionic Liquids in an Aqueous Two-Phase System Extraction

**Figure 2:**
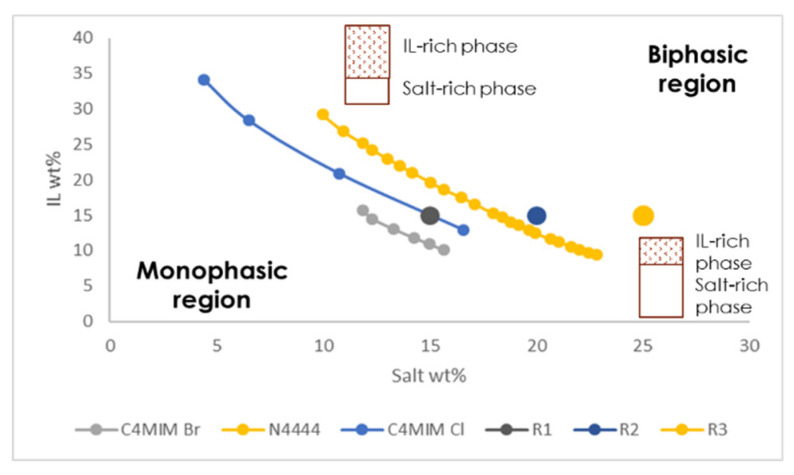
Binodal curve for the determination of weight ratio of IL, salt buffer (Na_2_CO_3_ or Na_2_CH_3_CO_2_) and water to produce biphasic systems. R1, R2 and R3 display the composition of the ATPS made with [C4Mim]Br^−^, [C4Mim]Cl^−^ and [N4444]Br^−^, respectively.

Ternary mixtures within the biphasic region were prepared for each IL-ATPS and consisted of either (1) 15 wt.% of [C4Mim]Br^−^, 15 wt.% Na_2_CO_3_ and 70 wt.% of the aqueous solution of pigmented fungal broth; (2) 15 wt.% of [C4Mim]Cl^−^, 20 wt.% Na_2_CO_3_ and 65 wt.% pigmented fungal broth; and (3) 15 wt.% of [N4444]Br^−^, 25 wt.% NaCH_3_CO_2_ and 60 wt.% pigmented fungal broth. The final volumes of the reaction mixtures were about 5 mL (final total weight of 5 g), with 0.75 g of the corresponding ionic liquid for 1 g of lyophilized fungal broth resuspended in corresponding weights of water and salt (i.e., 2.5 g of water + 0.75 g of Na_2_CO_3_, 2.25 g of water + 1 g of Na_2_CO_3_ or 2 g of water + 1.25 g of NaCH_3_CO_2_, respectively). The ternary mixtures’ compositions were chosen based on the phase diagrams of each IL investigated (Figure 2). The extraction step was performed as follows: all components (lyophilized pigment broth previously obtained by fermentations, salts, water and IL) were mixed together, vigorously stirred and allowed to equilibrate for 30 min before centrifugation at 2455× *g* (4000 rpm) for 5 min. The mixture was then allowed to decant overnight (16 h). The next day, each fraction of the mixtures was carefully separated, weighted out and its volume was measured. These values were used to calculate the coefficient of partition of pigments (K_pigment_), the phase ratio (R) and the selectivity of type of pigments ratio (S_pigment_), as well as the efficiency of extraction (Effp%), as described in the following sections.

The recovery of the pigments extracted from the IL phase was carried out by resuspending the previously collected IL phase in concentrated aqueous salt solution and adjusting the pH to 13. The mixture was stirred and left to stand for 30 min before being centrifuged at 2455× *g* (4000 rpm) for 5 min and allowed to decant overnight (16 h). Similar measurements of weight, volumes and absorbance were carried out and the efficiency of pigment recovery was calculated as described below.

### 2.5. Determination of the Coefficients of Partition of Pigments (K_pigment_)

After the extraction steps, samples of both IL-rich and salt-rich phases were diluted in distilled water and the pigment content of each phase was estimated by measuring the absorbance using a UV spectrophotometer (UV-1800, Shimadzu Corporation, Tokyo, Japan) at 490, 470 and 400 nm corresponding to red, orange and yellow hues, respectively. Relative repartitions (K_pigment_) of each type of pigment (red, orange and yellow) were calculated according to the following Equations (1)–(3):

K_red_ = (A490_IL_ * FD_IL_)/(A490_salt_ * FD_salt_) (1)

K_orange_ = (A470_IL_ * FD_IL_)/(A470_salt_ * FD_salt_) (2)

K_yellow_ = (A400_IL_ * FD_IL_)/(A400_salt_ * FD_salt_) (3)

where K_pigment_: coefficient of partition of the target shade of pigment (red, orange or yellow pigments); A_IL_: absorbance (mAU) of the IL phase sample at the wavelength considered (490, 470 or 400 nm); FD_IL_: dilution factor of the IL phase sample; A_salt_: absorbance (mAU) of the salt phase at the wavelength considered (490, 470 or 400 nm); FD_salt_: dilution factor of the salt phase.

### 2.6. Determination of the Selectivity of Type of Pigments Ratio (S_pigment_)

In order to evaluate the higher selectivity of ILs tested against the isolation of either red, yellow or orange pigments, the selectivity of type of pigments ratio (S_pigment_) was determined as follows:

S_red/yellow_ = K_red_/K_yellow_ (4)

S_red/orange_ = K_red_/K_orange_ (5)

where S_red/yellow_: selectivity ratio of red to yellow pigments extracted; K_red_: coefficient of partition of the red pigments extracted; K_yellow_: coefficient of partition of the yellow pigments extracted; Sr_ed/orange_: selectivity ratio of red to orange pigments extracted; K_orange_: coefficient of partition of the orange pigments extracted.

### 2.7. Determination of the Extraction Efficiencies (Eff%)

The efficiency of the direct extraction was determined according to the following formula:

EffD% = 100 × [K_pigment_/(1 + (1/R))] (6)

where EffD%: efficiency of the direct extraction; K_pigment_: coefficient of partition of the target pigments extracted; R: phase ratio between the IL- and salt-rich phase volumes.

The phase ratio (R) was calculated using the following equation:

R = V_IL_/V_salt_ phase (7)

where R: phase ratio; V_IL_: volume (mL) of the IL phase sample; V_salt_: volume (mL) of the salt phase.

## 3. Results

### 3.1. Determination of Biphasic Systems Using Binodial Curves

In the search for alternative extraction systems that are less hazardous for the environment and for human health, the properties of two different families of ionic liquids, namely the imidazolium- and the tetra-alkyl ammonium-based core families, were investigated specifically for extracting mixtures of fungal pigments. Subsequently, three IL-ATPSs, including [C4Mim]Br^−^ + Na_2_CO_3_, [C4Mim]Cl^−^ + Na_2_CO_3_ and [N4444]Br^−^ + NaCH_3_CO_2_, were investigated to evaluate their ability to selectively extract and detect mixtures of fungal pigments with different colors from fermentation broths [25]. Fungal fermentation broths are complex biological matrices containing various complex compounds, from proteins to lipids and other excretable secondary metabolites, including fungal pigments. Such diversity represents a burden for the development of fast, efficient and cost-effective detection and extraction processes, for which IL-ATPSs are expected to offer realistic options [19,21]. A brief description of the overall extraction process is shown in Figure 1. In this study, IL-ATPSs were applied to a lyophilized pigmented fermentation broth where the fungal pigments we are searching for (e.g., polyketide-derived pigments such as azaphilone red compounds, etc.) should have hydrophilic profiles.

Imidazolium cores have a stronger interaction with water than tetra-butyl ammonium and, consequently, tend to attract more hydrophilic systems. The combination with different anions and salts has an impact on the final overall hydrophilic/hydrophobic profile of the ionic liquid-rich phase, and this can be assessed by the determination of the binodal curves of each system [37,38,39]. Thus, binodal curves were produced for each of the IL-ATPSs in order to determine which ionic liquid/salt ratio was needed to generate a biphasic system. The resulting curves were produced using the Cloud Point titration method [28,36] and are displayed in Figure 2. We designed three IL-ATPSs that have in common the same ionic liquid content (15%), with the underlying strategy to avoid a large amount of ILs to be used due to both their cost and environmental impact. Although ionic liquids are considered as more ecological extraction solvents because of their higher extraction capacities for smaller volumes and recyclability, their final discard remains a grey area with regard to respect for the environment [14]. The corresponding amount of salt to be added for each specific ionic liquid system was established based on the binodal curves. Because clear and stable two-phase systems are desired, the less ATPS-forming a system is, the higher the amount of added salt (% *w*/*w*) was, in the limit of 25% *w*/*w* of the system. Both salts, sodium carbonate and sodium acetate, were adopted on the basis of their pH-buffering ability, as well as their biodegradability and lack of toxicity [40,41].

As shown in Figure 2, the phase-forming ability of the different ionic liquid systems can be ranked in the following order: [C4Mim]Br^−^ > [C4Mim]Cl^−^ > [N4444]Br^−^. From the binodal curves, surprisingly, the most ATPS-forming systems were the imidazolium-based ATPS. Due to its longer alkyl chains, [N4444]Br^−^ was expected to be the most prone to generate a two-phase systems. Among the systems investigated here, imidazolium-based ionic liquids were the least ATPS-forming options against tetra-alkyl ammonium-based ionic liquids, when taken under the same conditions (i.e., combination with the same anion and chaotropic/kosmotropic salts). Between the two classes of imidazolium-based ionic liquids chosen here, the only difference is the coupled anion (the salt being identical). [C4Mim]Br^−^ showed higher ATPS-forming capacity than [C4Mim]Cl^−^, and this is in line with previously reported results, where it was observed that bromide-based IL had greater aptitude to form ATPS than its chlorine anion-linked counterpart [37,42], when used under the same conditions (salts and ionic liquid core). This can be explained because bromide anions are more chaotropic than chlorine anions. The [N4444]Br^−^ system involves a different salt, namely an organic salt of sodium acetate. A previous study comparing the effect of the salt on the ability to generate a two-phase system reported sodium carbonate, an inorganic salt, as significantly inclined to form biphasic systems [37]. It is then reasonable to assume that the lower ability of [N4444]Br^−^ to form an ATPS, despite its longer alkyl chains, is mostly due to its combination with the organic salt of sodium acetate [43]. 

Those systems and their variation in profiles and behaviors have provided an unusual method for the selective extraction of pigments produced by submerged cultures of filamentous fungi from their fermentation broth. Indeed, we seek to selectively extract hydrophilic polyketide-derived pigments from ionic liquid-phase mixtures; therefore, we are investigating relatively hydrated ionic liquid phases that are still capable to form stable ATPSs.

### 3.2. Effect of the Ionic Liquids Aqueous Two-Phase System Extraction in the Partitioning of Fungal Pigments

**Figure 3:**
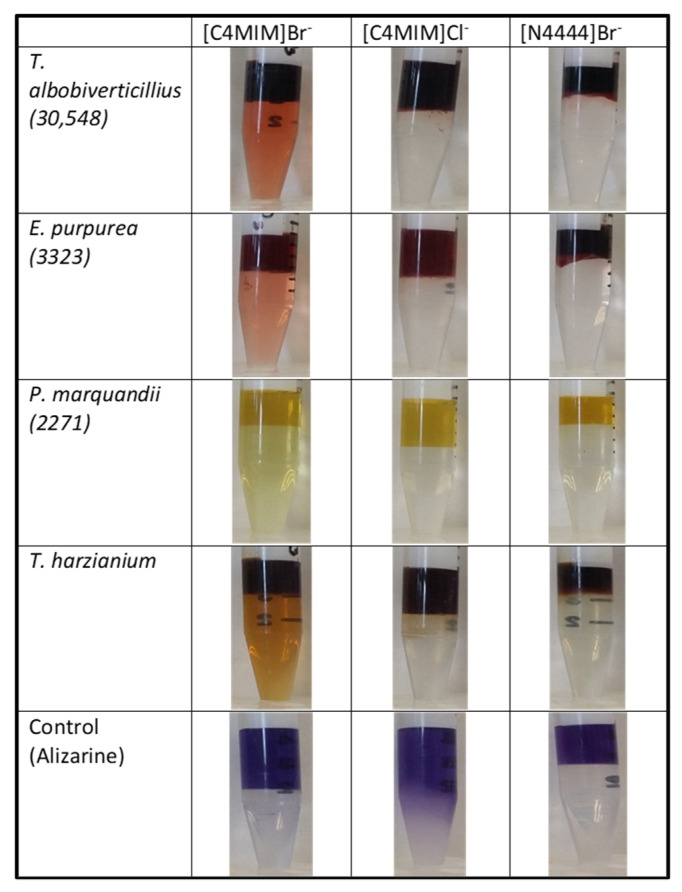
Visual aspect of the first step of extraction from the different IL-ATPSs tested and their extraction abilities on fungal fermentation broth and pure pigment control (alizarin). Lyophilized colored fermentation broths were mixed with the corresponding ionic liquid and salt. The mixtures were allowed to equilibrate for 30 min before centrifugation for 5 min at 4000 rpm and overnight decantation. Both ionic liquid-rich and salt-rich phases were collected and absorbances were measured. Top phase: ionic liquid-rich phase where most of the pigments migrated; Bottom phase: salt-rich phase.

In a previous published study, we identified the following four fungal strains: *T. albobiverticillius* of marine origin (deep red) and three strains of terrestrial origin, i.e., *E. purpurea* (red), *P. marquandii* (yellow) and *T. harzianum* (yellow-brown), based on their capacities to produce high amounts of polyketide-derived pigments in fermentation broth. One of the main limiting steps in the bioproduction of pigments is their inefficient and cost-effective extraction. We selected the aforementioned fungal strains to investigate the potential of IL-ATPS extraction for the selective detection and extraction of fungal pigments with different colors. Currently, there is a specific need for natural alternatives for red pigments for industrial use. We therefore focused our work on the potentialities of extracting fungal red pigments by IL-ATPS selected here and, more particularly, on the azaphilone-like red pigments from the fermentation broth of the marine-derived filamentous fungi *T. albobiverticillius* 30548, which was collected on the west coast of Réunion Island. Both tetra-butyl ammonium and imidazolium ionic liquids were chosen because they are considered more compatible (and less disruptive) with biomolecule structures and have proven large spectra of applications. Moreover, previous studies reported successful partitioning and recovery of hydrophobic pigments such as carotenoids using IL-ATPSs with similar polarity profiles as the target compounds [21,22,40,44,45]. Thus, the same strategy was applied here by investigating IL-ATPSs with greater hydrophilic profiles for the purpose of better azaphilone-like-pigment isolation. Based on these initial properties, it was assumed that the more hydrophilic the IL top phase, the higher the pigment partition coefficient and, consequently, the better the extraction would be. The results of the first direct extraction are shown in Figure 3. Globally speaking and across all three ATPSs tested here, the results of extraction showed that pigments tend to preferentially migrate towards the top ionic liquid-rich phase. Unanimously, [C4Mim]Br^−^ with 15 wt.% Na_2_CO_3_ exhibited the smallest pigment partition in the four pigment-producing strains. Indeed, there was a significant loss of pigments remaining in the aqueous salt-rich phase, as shown in Figure 3. On the other hand, both systems, i.e., [C4Mim]Cl^−^ + 20 wt.% Na_2_CO_3_ and [N4444]Br^−^ + 25 wt.% NaCH_3_COO, showed significantly higher pigment migration in the IL top phase, confirming their greater potential as an extraction system for hydrophilic fungal polyketide-derived pigments of various colors (red, orange and yellow).

**Figure 4:**
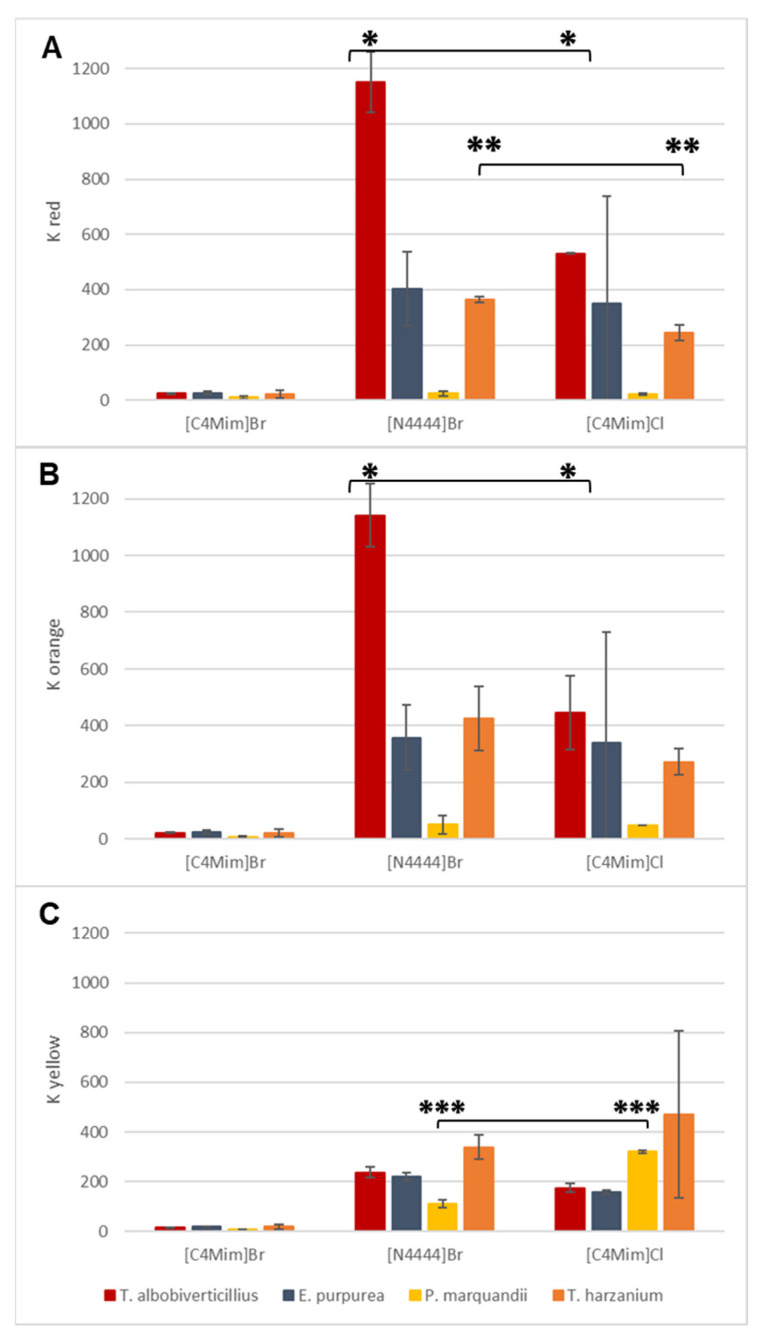
Partition coefficients of red (490 nm) (**A**), orange (470 nm) (**B**) and yellow (400 nm) (**C**) pigments for each strain in the four IL-ATPSs tested (*, ** and *** stand for statistical significance based on *p*-values < 0.05 performed for the same strain treated with different IL-ATPSs, *n* = 3).

**Figure 5:**
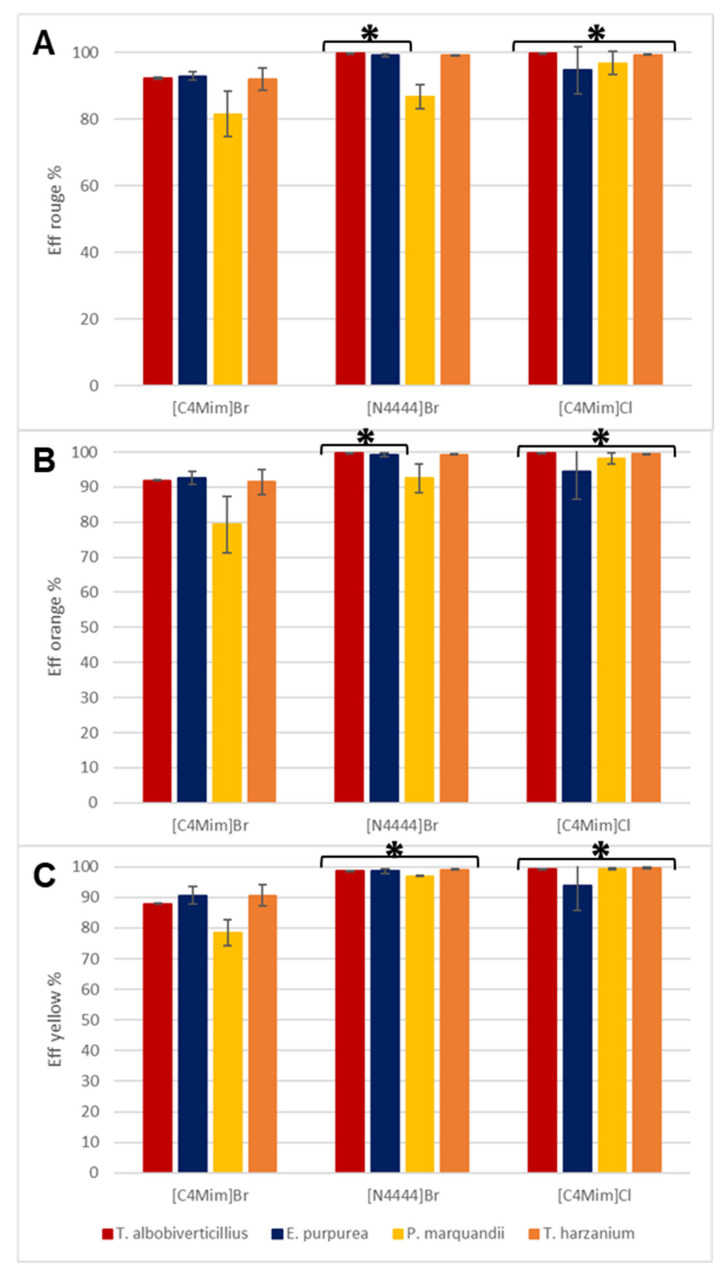
Direct extraction efficiency of red (490 nm) (**A**), orange (470 nm) (**B**) and yellow (400 nm) (**C**) pigments for each strain in the four IL-ATPSs tested (* indicate *p*-values < 0.05, *n* = 3).

The coefficients of partition of distinct pigment shades (K_red_, K_orange_ and K_yellow_) express the preference of each type of pigment to migrate in ionic liquid-rich phase. They were then calculated for each system and are displayed in Figure 4. In line with previous observations, [C4Mim]Br^−^ + 15 wt.% Na_2_CO_3_ resulted in the lowest K values for all pigment shades for the four strains (Figure 4) and it was concluded as a non-suitable system for our purpose. Although the partition coefficient values obtained from the two different remaining ATPSs on the same fermentation broths suggested that the design of the IL-based ATPSs has an impact on pigment migration, it is important to consider that the composition in pigments and their respective polarity profiles will have an impact on the efficiency of the overall extraction of the color shade. We therefore investigated two red-pigment-producing fungal strains, namely *T. albobiverticillius* (30548) and *E. purpurea* (3323), which were submitted to the same extraction process. Interestingly, [N4444]Br^−^ + 25 wt% NaCH_3_COO statistically showed significantly better pigment partition coefficients for red to orange pigments from *T. albobiverticillius* than those from *E. purpurea* and *T. harzianum*. On the other hand, the extraction efficiency was equally as good for both strains using [N4444]Br^−^- and [C4Mim]Cl^−^-based ATPSs (Figure 5). These observations led to the conclusion that quarterly ammonium-based IL-ATPS (i.e., N4444]Br^−^) has better extraction capacities towards azaphilone-like red pigments. Moreover, it was noticed that the extraction yields tended to be less repeatable across the replicate experiments performed on pigments from *E. purpurea* for both [N4444]Br^−^ and [C4Mim]Cl^−^ based ATPSs. This led to the conclusion that the composition of the pigment mixture highly influenced the efficiency of one system. Previous studies have concluded on the production of azaphilone-like red compounds mainly by *T. albobiverticillius,* suggesting mostly hydrophilic molecules. [26]. The binodal curve initially established here highlighted the [N4444]Br^−^ ATPS as the most hydrophilic system amongst our ATPSs, which could explain why and how this specific system appeared to be a more appropriate extraction agent for an apparent homogenous mixture of reddish hydrophilic pigments.

In parallel, the repartition coefficient for yellow pigments remained drastically smaller than repartition coefficient for red, K_red,_ across all ATPSs as well as amongst all fungal strain productions. Even the bright-yellow-pigment-producing fungal strain, namely *P. marquandii*, did not generate partition coefficient values as high as red-pigment-producing fungal strains (i.e., *T. albobiverticillius* and *E. purpurea*) for red to orange pigments. Such limited results for yellow compounds could be explained by the fact that reddish and orangish pigment-producing fungal strains may simply not produce many actual yellow molecules. In the case of the strong yellow-producing strain of *P. marquandii*, although the pigments clearly migrated to the ionic liquid-rich phase, the raw data of absorbance measured at 400 nm (yellow typical wavelength) were globally four times weaker in reddish strains, suggesting a smaller inherent yellow-pigment-production compared to red. Nevertheless, as shown in Figure 4C, no statistically significant differences were observed in yellow compound extractions from one IL-ATPS to another when used on broth containing a mixture of pigments produced by *T. albobiverticillius*, *E. purpurea* or *T. harzianum*. On the contrary, on the yellow-producing strain, namely *P. marquandii,* a statistically significantly better extraction capacity (i.e., higher K_yellow_) was observed with [C4Mim]Cl^−^.

Thus, from this first round of extraction, [N4444]Br^−^ appeared as the more appropriate system amongst the ones investigated herein for consistent and efficient extractions of azaphilone-like red compounds from fungal resources. The yellow polyketide-based secondary metabolites produced by the investigated fungal strains were not the most preferred pigments extracted by either [N4444]Br^−^ or [C4Mim]Cl^−^; however, [C4Mim]Cl^−^ showed relatively good robustness and efficiency with respect to the extraction of these yellow compounds (such as yellow azaphilone pigment T22 from *T. harzianum* and yellow sorbicillinoids (hexaketide compounds) from *P. marquandii*). It is worth mentioning that it seems more suitable to use this system on a strain generating a strongly yellow broth, as more inconsistent extraction results were observed on the yellow-brown mixture produced by *T. harzianum*. Thus, further optimizations, in particular on salt combinations and/or ratio thereof, could lead to better detection and extraction capacities.

### 3.3. Selectivity of the IL-ATPS for Red Pigments in Various Fungal Broths

**Figure 6:**
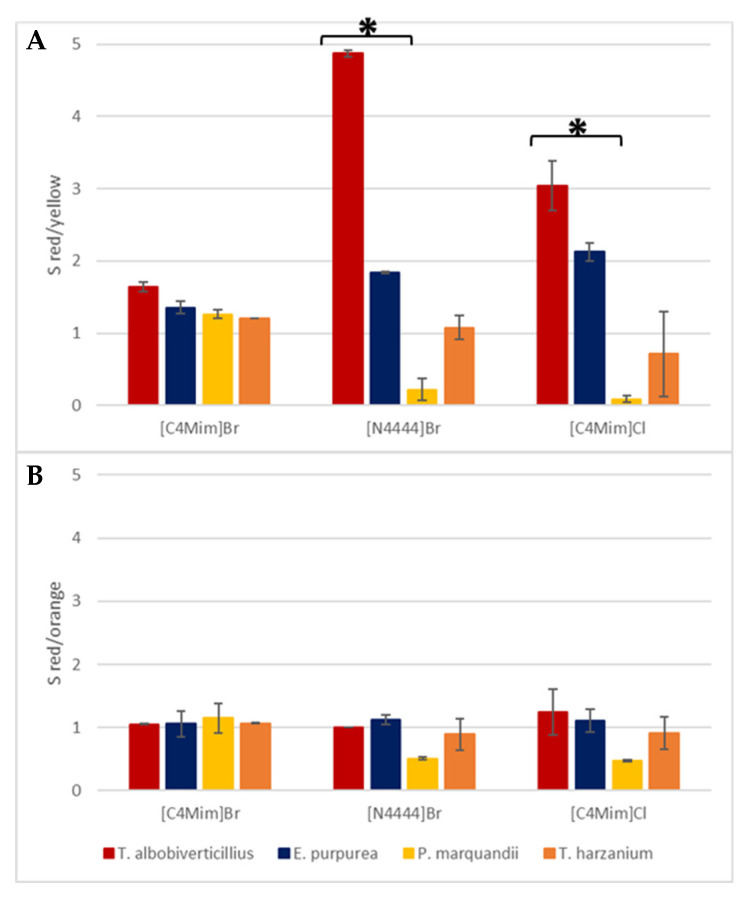
Extraction selectivity of red pigments over yellow pigments, S_red/yellow_ (**A**) and over orange S_red/orange_ (**B**) pigments for each strain in the four IL-ATPSs tested (* indicates *p*-values < 0.05, *n* = 3).

As mentioned earlier, the selective isolation of red pigments from other fungal pigments and cellular components is the main goal of this study, due to the higher potential market of red colorants for industrial application [3,4,25]. To further determine the ability of each IL-ATPS to detect and isolate red colorants, two selectivity parameters were calculated as the ratio of the partition coefficients of red pigments to other fungal pigments and are displayed in Figure 6.

Overall, in red-producing strains, the selective extraction of red over yellow co-produced colorants was strongly favored, as suggested by the high S_red/yellow_ values (i.e., ratio S_red/yellow_ >>1) reported across the IL-ATPSs considered. In particular, we reported a statistically significantly better selectivity of red pigments’ extraction from *T. albobiverticillius* when using [N4444]Br^−^ compared to [C4Mim]Cl^−^, while no significant difference was observed from *E. purpurea*. This suggested that the migration and selectivity of mixtures of pigments are greatly impacted by the features of the ILs used. As far as we can see and without knowing the detailed composition of pigments in the different mixtures, we confirmed that the [N4444]Br^−^-based ATPS was the most suitable extraction system for the selective isolation of red colorants across different hue-producing strains. On the other hand, little or no selection occurred between red and orange pigments as shown in Figure 6B and in all IL-ATPSs and studied strains. Indeed, we reported a selective extraction ratio for red/orange pigments close to 1 for all selected strains (Figure 6B). We also noticed small distinction between red and orange or yellow colorants in all the IL-ATPSs used on *T. harzianum* (Figure 6A,B), suggesting a strong likeness of concomitant extraction of red and orange pigmented molecules from the fermentation broth showing a mixture of pigments of different shades. These observations led to the conclusion that our [N4444]Br^−^ system showed its highest potential under the conditions investigated here when applied on single-tone broth.

### 3.4. Reverse Extraction of the Fungal Pigments from the IL-ATPSs

The main objective of this study was to explore the potential of IL-ATPSs for the selective detection and extraction of red fungal pigments from a mixture of colored molecules. We succeeded in showing promising detection and extraction efficiencies of the [N4444]Br^−^ system for azaphilone-based compounds. This satisfied our initial scientific question. To further explore the potential applications of this technique, we investigated the back-extraction efficiency of the pigments from the IL system using a previously reported method. This was carried out with the purpose of rapidly estimating how straightforward and repeatable back extractions from IL-based systems across different types of biological matrixes are. Although several publications have reported the benefits of using IL-based extraction systems for the isolation of bioactive compounds [46], very few have addressed the corresponding challenges to develop efficient extraction of the said bio-compounds from IL-based solvents (referred to as back-extraction) [23,47]. Tan and coworkers [43] succeeded in back-extracting the hydrophilic anthraquinones of aloe-emodin from their IL-ATPS with [C4Mim]BF4 by adding an alkaline salt solution onto the IL-rich phase containing the pigment. The same approach was applied here using an alkaline (pH 13) solution of the respective salts initially used for each system, which were added to the separated IL-rich phase, shaken and left for decantation overnight. The visualization of the back-extraction steps before and after the alkalinization of the salt solution is shown in Figure S1. The visual aspects of the extracts (Figure S1) indicate that the reverse extractions remained extremely limited. The increase in pH up to 13 using drop-wise addition of NaOH did not allow a better recovery of any of the pigments tested under our experimental conditions. After the addition of the salt solution and before the alkalinization of the system, the phase separation between the IL- and salt-rich phases became blurry, especially for the [C4Mim]Cl^−^ system (Figure S1). Interestingly, in the [C4Mim]Cl^−^ and [C4Mim]Br^−^ ATPSs, before the pH increase, a slight repartition of the pigments in the salt phase for the red-producing strains (*T. albobiverticillius* and *E. purpurea*) and the brown-producing strain (*T. harzianum*) was observed. The increase in pH appeared to have counteracted the recovery of the pigments in the salt-rich phase, whilst further improving the partition in the IL-rich phase, as can be seen in particular with the clarification of the salt-rich phase in the case of the [C4Mim]Cl^−^ across the four fermentation broths tested (Figure S1).

The overall efficiencies and abilities of recovery of red pigments from the IL-ATPS after application of an alkaline salt solution are displayed in Figure S2. We concluded that the recovery of the pigments from any of the fermentation broth tested was very poor under these conditions. The highest overall recovery was obtained from the fermentation broth from *E. purpurea*, with a final recovery of about 20% (Figure S2B). We were also forced to conclude that we could not extract back, from the IL phase, the dark red pigments excreted by either *T. albobiverticillius* or *E. purpurea* because the pigments did not migrate to the alkaline salt-rich phase. Curiously, it seemed that increasing the pH reduced the migration of the pigments, which was in contradiction with the results obtained by Ventura et al. [40] on hydrophilic anthraquinone of aloe-emodin. However, this can be put into perspective by the fact that, firstly, we do not know the exact hydrophilic/hydrophobic equilibrium of all the fungal pigments produced in the fermentation broths, and secondly, we directly applied these IL-ATPSs onto complex fermentation matrices. Then, plenty of other metabolites were likely to interfere in the selective extraction of pigmented compounds. Globally speaking and despite the low overall recovery values, we could notice a statistically significant difference in the [N4444]Br^−^-based IL-ATPS on the extraction, back-extraction and overall recovery of red pigments. In conclusion, we were able to recover more red pigments from the fermentation broth of the second most red-pigment-producing strain, namely *E. purpurea,* by using the [N4444]Br^−^ ATPS.

To date, ionic liquids are considered to be a sustainable means of extraction, mainly because of their very high extraction power, perfectly illustrated here, revealing a promising potential as cost-effective, profitable and less hazardous solvents. However, it is important to recall that ionic liquids require heavy organic chemical synthesis, which can be counterintuitive against their reputation of being new-age green solvents. However, few arguments can be given here in favor of ionic liquids’ green profile against conventional extraction solvents. First, the already well-known higher extraction power and recyclability of ionic liquids are their dire assets, as previously said. Second, a recent paper estimated and compared the environmental sustainability between a similar IL-based extraction system applied to carotenoid recovery and a conventional solvent extraction method (using acetone and ethanol) by calculating environmental factor (E-factor) and carbon footprint parameters [45]. The E-factor and carbon footprint calculations included all greenhouse gas (GHG) emissions from the production of all chemicals and the water and electricity consumed during the process. It was concluded that IL-based extraction offered a significantly reduced environmental impact compared to conventional solvents. Perspective must be taken though, as efficient recycling of both IL materials and water is paramount for ionic liquids to be properly referred to as green solvents.

The poor overall recovery obtained here highlights essential points of improvements to be made while perfectly illustrating how much case-by-case optimization is needed to use ionic liquids for detection and extraction purposes. It is worth mentioning that there are various options to explore in order to optimize back-extractions and enable multiple re-use of ionic liquids, such as using a different salt or polymer solutions as antisolvents, changing temperatures and applying ultrasounds or microwaves and a combination thereof [48]. Depending on the type and sensitivity of the target compounds trapped in IL phase, distillation or even adsorption can also be used. However, these options need to be carefully considered based on the additional energy, water, time and specific equipment expenditures they would require. However, this point was not in the scope of this paper.

## 4. Conclusions

Our findings suggested that the combination of an ATPS extraction system using ionic liquids as greener extraction solvents is a possible tool for the extraction and detection of colored molecules from the fermentation broth of filamentous fungi [49]. Among the IL-based systems investigated here, the [N4444]Br^−^ based system appeared to have the best ability to extract fungal pigments, with putative higher affinity for red-orange azaphilone-like pigments. Thus, the IL-ATPS consisting of 15% [N4444]Br^−^ and 25% Na_2_CH_3_CO_2_ was found to be a promising solvent that is worth further investigation to expand its potential applications. Indeed, at this point, the most appropriate use of this system would be for the detection of red azaphilone-like pigments. More studies and optimizations, especially at the back-extraction level, are needed to use this [N4444]Br^−^ system as a powerful extraction technique. Thus, further studies would have to be carried out at larger scales in order to evaluate the obstacles and challenges associated with efficient target molecule recoveries and ionic liquids’ recyclability.

## Figures and Tables

**Table 1:** Description of the selected ionic liquids (ILs) associated with the kosmotropic salt buffer, enabling a diphasic system.

ILs	Salt	Structure
[C4Mim]Br^−^ 1-Butyl-3-methylimidazolium bromide	Na_2_CO_3_ Sodium carbonate (Inorganic)	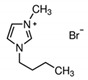
[C4Mim]Cl^−^ 1-Butyl-3-methylimidazolium chloride		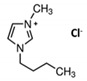
[N4444]Br^−^ Tetrabutylammonium bromide	NaCH_3_CO_2_ Sodium acetate (Organic)	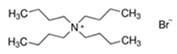

## Supplementary Materials

The following are available online at https://www.mdpi.com/2309-608X/6/4/375/s1, Figure S1: Visual aspect of the reverse extraction steps for each different IL-ATPS tested before (pH = 5) and after adjusting the pH (pH = 13), Figure S2: Back extraction efficiency (**A**) and overall efficiency (**B**) of red pigments extraction for each strain in the 4 IL-ATPS tested (*n* = 3).
