# Supplementary material for: Aqueous Two-Phase System Extraction of Polyketide-Based Fungal Pigments Using Ammonium- or Imidazolium-Based Ionic Liquids for Detection Purpose: A Case Study
Source: J Fungi (Basel). 2020 Dec 18;6(4):375. doi: 10.3390/jof6040375 (PMC7766805; doi:10.3390/jof6040375)
Supplement: Supplementary file 1 [file jof-06-00375-s001.pdf]

Journal of *Fungi*

Article

Aqueous two-phase system extraction of polyketide-based fungal pigments using ammonium or imidazolium-based ionic liquids for detection purpose: a case study

Juliana Lebeau [1], Thomas Petit [1,2], Mireille Fouillaud[1], Laurent Dufosse[1] and Yanis Caro [1,2]*

[1] Laboratoire de Chimie et de Biotechnologie des Produits Naturels (CHEMBIOPRO), Université de La Réunion, Sainte-Clotilde, F-97490, Reunion Island, France
[2] Département Hygiène Sécurité Environnement (HSE), Université de La Réunion – IUT La Réunion, Saint-Pierre, F-97410, Reunion Island, France
* Correspondence:Correspondence: yanis.caro@univ-reunion.fr (Y.C.)

Supplementary Materials

Figure S1: Visual aspect of the reverse extraction steps for each different IL-ATPS tested before (pH=5) and after adjusting the pH (pH=13). The previously collected IL-rich phases were resuspended in their corresponding concentrated aqueous salt solution and the pH was adjusted to 13 by the dropwise addition of NaOH. The mixtures were left to equilibrate for 30min before centrifugation for 5min at 4000rpm and overnight decantation. Both IL-rich and salt-rich phases were collected again and absorbances measured. Top phase: IL-rich phase; Bottom phase: salt-rich phase, Figure S2: Back extraction efficiency (A) and overall efficiency (B) of red pigments extraction for each strain in the 4 IL-ATPS tested (n=3).

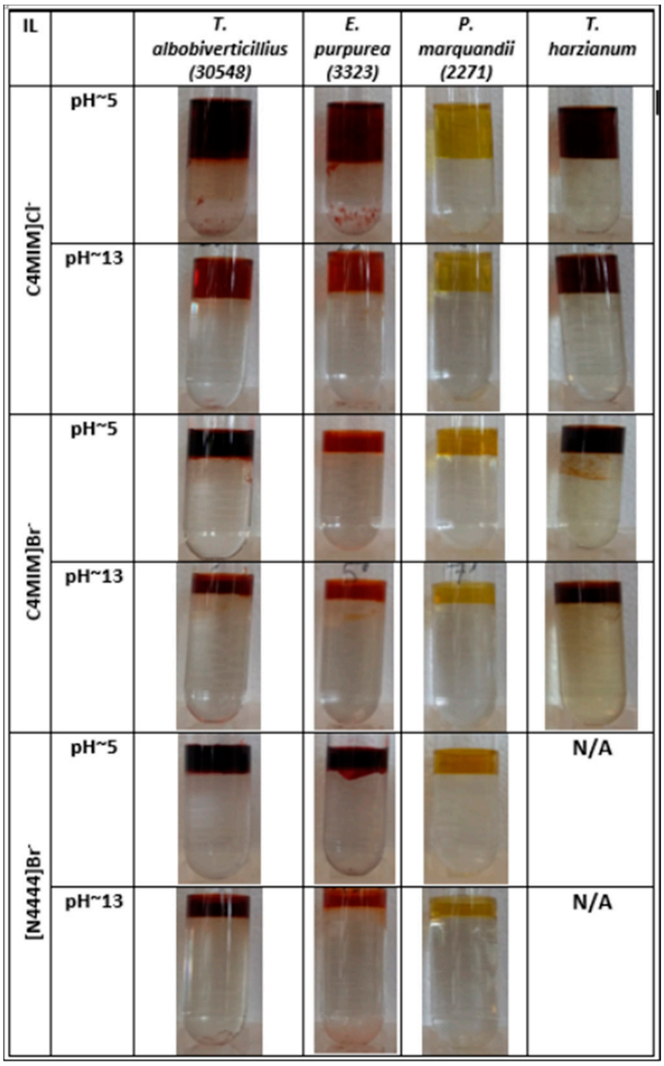

Figure S1. Visual aspect of the reverse extraction steps for each different IL-ATPS tested before (pH=5) and after adjusting the pH (pH=13). The previously collected IL-rich phases were resuspended in their corresponding concentrated aqueous salt solution and the pH was adjusted to 13 by the dropwise addition of NaOH. The mixtures were left to equilibrate for 30min before centrifugation for 5min at 4000rpm and overnight decantation. Both IL-rich and salt-rich phases were collected again and absorbances measured. Top phase: IL-rich phase; Bottom phase: salt-rich phase.

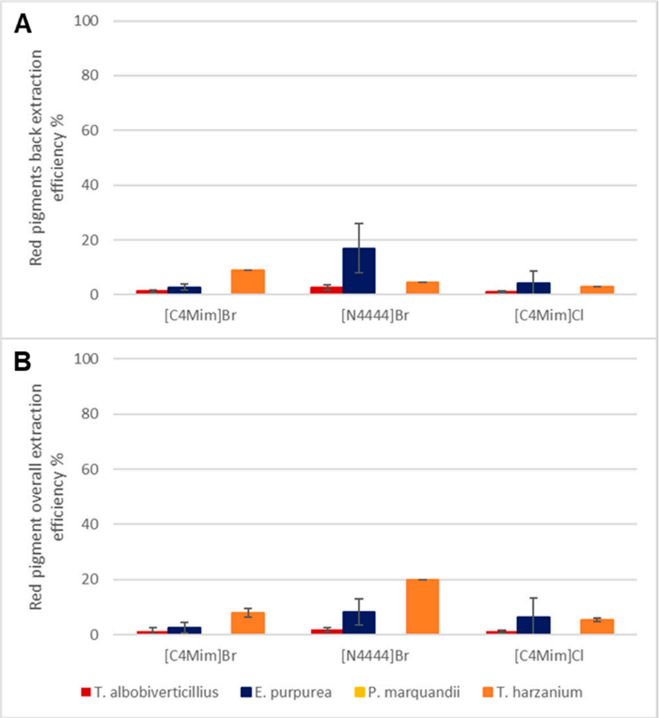

Figure S2. Back extraction efficiency (A) and overall efficiency (B) of red pigments extraction for each strain in the 4 IL-ATPS tested (n=3). The overall (direct + reverse) extraction efficiency (Eff tot%) was calculated as follows:

$$\text{Eff tot\%} = m_{\text{back extraction salt phase}} / m_o$$

with m_o: initial mass of the target pigments provided by the fermentative broth (expressed in terms of milliequivalent ($m_{eqv.}$) of polyketide pigments), $m_{\text{back extraction salt phase}}$: mass of the target pigments (in m_{eqv}. of polyketide pigments) recovered in the salt phase after the second extraction.
